# Peri-interventional neutrophil extracellular traps and immune surveillance in hepatocellular carcinoma: mechanisms, biomarkers, and recurrence after liver resection or thermal ablation

**DOI:** 10.3389/fonc.2026.1988923

**Published:** 2026-09-28

**Authors:** Hang Xu, Jiayang Yi, Wanwan Zhang, Shaojie Zhu, Zhigang Liu

**Affiliations:** 1 Graduate School, Wannan Medical University, Wuhu, Anhui, China; 2 Department of Hepatobiliary and Pancreatic Surgery, The Second People’s Hospital of Wuhu, Wuhu, Anhui, China; 3 Department of Rheumatology and Immunology, The First Affiliated Hospital of Wannan Medical University, Wuhu, Anhui, China

**Keywords:** antitumor immunity, biomarkers, hepatocellular carcinoma, ischemia-reperfusion, liver resection, neutrophil extracellular traps, thermal ablation, tumor recurrence

## Abstract

Recurrence remains common after curative-intent liver resection or thermal ablation for hepatocellular carcinoma (HCC). These interventions transiently remodel the hepatic and treatment-bed immune microenvironment through overlapping but distinct processes, including tissue injury, vascular and coagulation activation, neutrophil recruitment, and, particularly during resection, hepatic ischemia-reperfusion. We organize the evidence using a peri-interventional two-component heuristic: chronic liver disease and tumor-associated inflammation may shape baseline susceptibility, whereas acute treatment injury may trigger intervention-specific neutrophil extracellular trap (NET) accumulation. Experimental studies across liver-intervention and cross-cancer models link NETs to impaired CD8^+^ T-cell immunity, tumor-cell retention, extracellular-matrix remodeling, and metabolic adaptation. Patient-linked evidence remains limited and largely associative; much of the mechanistic framework therefore relies on colorectal liver metastasis and cross-cancer models. Translational studies should distinguish monitoring, pharmacodynamic, prognostic, and predictive applications and prioritize serial MPO–DNA as the best-supported circulating peri-interventional candidate, with NE–DNA or H3Cit-based measures as supportive assays and spatial validation where feasible. Candidate preclinical approaches include limiting NET formation, degrading established NET structures, and disrupting NET–target-cell signaling. NET–checkpoint combinations remain preclinical hypotheses, particularly because current phase III adjuvant ICI studies have not established postoperative HCC benefit. Because NET and neutrophil functions are context dependent, prospective studies should define intervention-specific NET kinetics, immune correlates, target engagement, recurrence phenotype, and safety.

## 1 Introduction

Liver resection and thermal ablation offer curative-intent treatment for selected patients with early hepatocellular carcinoma (HCC), yet local progression and intrahepatic recurrence remain frequent (1, 2). Outcome reflects tumor biology and treatment quality, but the procedure itself also creates a short-lived inflammatory state. Surgical manipulation, hepatic ischemia-reperfusion, and thermal injury release damage-associated molecular patterns, activate vascular and coagulation pathways, and recruit neutrophils (3, 4). During this period, the treated liver may contain microscopic residual disease, surviving peri-ablational tumor cells, occult micrometastases, dormant disseminated cells, and circulating tumor cells (CTCs) (5–7). For clinical interpretation, we distinguish incomplete-treatment-related local progression; early intrahepatic recurrence, commonly within 2 years and more often reflecting residual or micrometastatic disease; extrahepatic metastasis; and late intrahepatic recurrence beyond approximately 2 years, which more often represents *de novo* multicentric HCC. These categories overlap biologically, but an acute peri-interventional NET response is more plausible for the first three than for late field carcinogenesis (1, 2).

Neutrophil extracellular traps (NETs) are extracellular chromatin structures associated with histones and granule proteins, including myeloperoxidase (MPO) and neutrophil elastase (NE) (8). Their release can occur through lytic and non-lytic programs; extracellular NET-associated material therefore does not, by itself, establish canonical peptidylarginine deiminase 4 (PAD4)-dependent NETosis (9, 10). We use NET formation, release, or accumulation unless a defined cellular program was demonstrated.

Recent reviews have examined perioperative NETosis across cancers, NET networks in HCC, spatiotemporal heterogeneity, and biomarker/therapeutic strategies (11–13). This Mini Review focuses on cancer immunity around HCC resection and thermal ablation. Where direct peri-interventional HCC evidence is lacking, hepatic-intervention and liver-metastasis studies serve as mechanistic bridges, with cross-cancer studies used only for otherwise unavailable mechanisms. Transarterial and radiation-based therapies are outside the scope because direct peri-interventional NET evidence is less developed. Throughout the text and **Table 1**, we label evidence as HCC-specific (HCC), hepatic-intervention without an HCC-specific oncologic endpoint (HI), liver-metastasis (LM), or cross-cancer mechanistic (XC). These labels describe source and context, not formal certainty, and are repeated at key extrapolation points.

**Table 1:** Sentinel studies informing peri-interventional NET biology, evidence calibration, and recurrence-relevant outcomes.

Context	Human evidence: n; timing/specimen; assay	Experimental component	Mechanism / key finding	Evidence domain	Most relevant phenotype / horizon	Critical limitation and citation
Surgical stress after CRLM resection	n=50 CRLM hepatectomy; POD1 serum; MPO–DNA ELISA (major n=35, minor n=15)	Mouse hepatic I/R metastasis model; DNase I / PAD4 perturbation	Higher postoperative MPO–DNA associated with shorter DFS; NET deposition promoted metastatic growth	LM	Early metastatic recurrence / dissemination	Observational human association; not primary HCC; single postoperative time point (14).
Hepatic ischemia–reperfusion	n=15 partial-hepatectomy patients; pre/POD1 peripheral neutrophils; P-SYK flow cytometry; mechanistic Co-IP subset n=4	Murine 70% warm I/R; SYK inhibition; MPO/NE/CitH3 readouts	Neutrophil SYK–PKM2–STAT3 plus macrophage SYK–NLRP3–IL-1β; experimental recurrence	HI	Early recurrence after I/R (experimental)	Human component assessed pathway activity, not recurrence (15).
Posthepatectomy regeneration / PHLF	Plasma n=99 (PHLF n=24), POD−1/POD1/POD5; CitH3/MPO/NE; tissue n=35 (PHLF n=10), fluorescence microscopy	Murine partial hepatectomy; DNase I	Excess NET accumulation associated with PHLF; NET degradation accelerated hepatocyte proliferation/regeneration	HI	Regenerative safety; not an oncologic recurrence phenotype	Not HCC-specific; informs safety rather than recurrence causality (16).
Clinical thermal ablation (MWA)	n=40 mixed liver cancers (24 primary/16 secondary); pre and 24 h; MPO–DNA n=40, H3Cit n=20	Mechanistic experiments performed with RFA, not MWA	MPO–DNA rose after MWA; fold increase greater with local progression	HI	Local progression / early recurrence	Mixed malignancies; independent prognostic value and margin/zone effects unresolved (17).
Insufficient RFA	CRLM n=167: iRFA n=41, complete RFA n=46, resection n=80; residual tumor defined within 4 months	Mouse iRFA models; neutrophil/PAD4 pathway perturbation	Residual CRLM induced CD177hi/PAD4hi neutrophils, NET-associated signaling and lung metastasis	LM	Incomplete-treatment local progression; extrahepatic metastasis	Persistent CRLM after iRFA cannot represent technically complete HCC ablation (18).
Perioperative hepatic I/R and T-cell exhaustion	CRLM resection: pre/POD1 serum MPO–DNA/sPD-L1 n=11; NET/PD-L1 neutrophil phenotyping n=3	Hepatic I/R metastasis models; DNase I or perioperative PD-L1 blockade	PD-L1-bearing NETs promoted functional/metabolic T-cell exhaustion	LM	Residual/micrometastatic growth; early recurrence	Small human mechanistic subset; not HCC-specific; no clinical treatment effect (19).
Surgery-associated metabolic adaptation	Paired preoperative/POD1 plasma functional assays; small patient subsets (n=5–10/group)	Cross-cancer laparotomy models; DNase/GSK484/PAD4/metabolic perturbation	NET-induced MYC–CD36–fatty-acid oxidation supported anoikis resistance	XC	Early survival of disseminated cells / metastasis	Human component was ex vivo functional plasma testing; not HCC-specific (20).
Liver-metastasis NK surveillance	Metastatic liver specimens n=20 and non-metastatic liver controls n=6; tissue immunofluorescence; public human scRNA-seq n=3/group	Breast-cancer liver-metastasis models; CCDC25/pathway perturbation	NET-DNA sensing by CCDC25 suppressed NK activating receptors via ILK–STAT3	LM	Metastatic dissemination to liver	Not HCC-specific and not peri-interventional (21).
HCC immune microenvironment	HCC tissue n=201 (CitH3/MPO IF) and serum n=60 (MPO–DNA ELISA); cross-sectional	Complementary HCC models; PAD4/TMCO6/TGF-β1 perturbation ± anti-PD-1	NET-DNA–TMCO6 suppressed CD8^+^ TCR–NF-κB signaling	HCC	HCC progression; mechanistic relevance to residual/early disease	HCC-specific but not linked to resection/ablation timing (22).
RFA-associated antitumor immunity	n=69 HCC; pre/post-RFA PBMC; IFN-γ ELISPOT and tetramer assays	None for NET mechanism	TAA-specific T-cell responses increased in 62.3% and associated with recurrence-free outcomes	HCC	Post-RFA recurrence (countervailing immune response)	NETs were not measured; supports non-uniform post-ablation immunity (23).

CRLM, colorectal liver metastasis; CTC, circulating tumor cell; HCC, hepatocellular carcinoma; HI, hepatic-intervention evidence without an HCC-specific oncologic outcome; iRFA, insufficient radiofrequency ablation; LM, liver-metastasis evidence; MPO, myeloperoxidase; MWA, microwave ablation; NE, neutrophil elastase; NET, neutrophil extracellular trap; PAD4, peptidylarginine deiminase 4; PHLF, posthepatectomy liver failure; POD, postoperative day; RFA, radiofrequency ablation; XC, cross-cancer mechanistic evidence. Evidence domains describe source and context rather than formal certainty-of-evidence or risk-of-bias ratings. Human sample sizes, sampling time, and assays are shown where reported; **Table 1** is intentionally selective rather than exhaustive.

To organize the discussion, we use a two-component heuristic rather than a causal two-hit model. Baseline susceptibility comprises pre-intervention host and tumor states—chronic liver disease/cirrhosis, tumor-associated inflammation, and platelet–coagulation activation—that may influence neutrophil recruitment or NET formation (24–27). Acute treatment injury comprises procedure-specific inflammatory stimuli generated by resection, ischemia–reperfusion, or thermal injury (15). This descriptive, measurement-oriented framework does not require two sequential insults, assume either component is necessary or sufficient, or presuppose synergy. Baseline state and post-treatment trajectory should therefore be measured separately; a low baseline marker may still be followed by a large injury-driven peak. **Figure 1** summarizes the framework.

**Figure 1:**
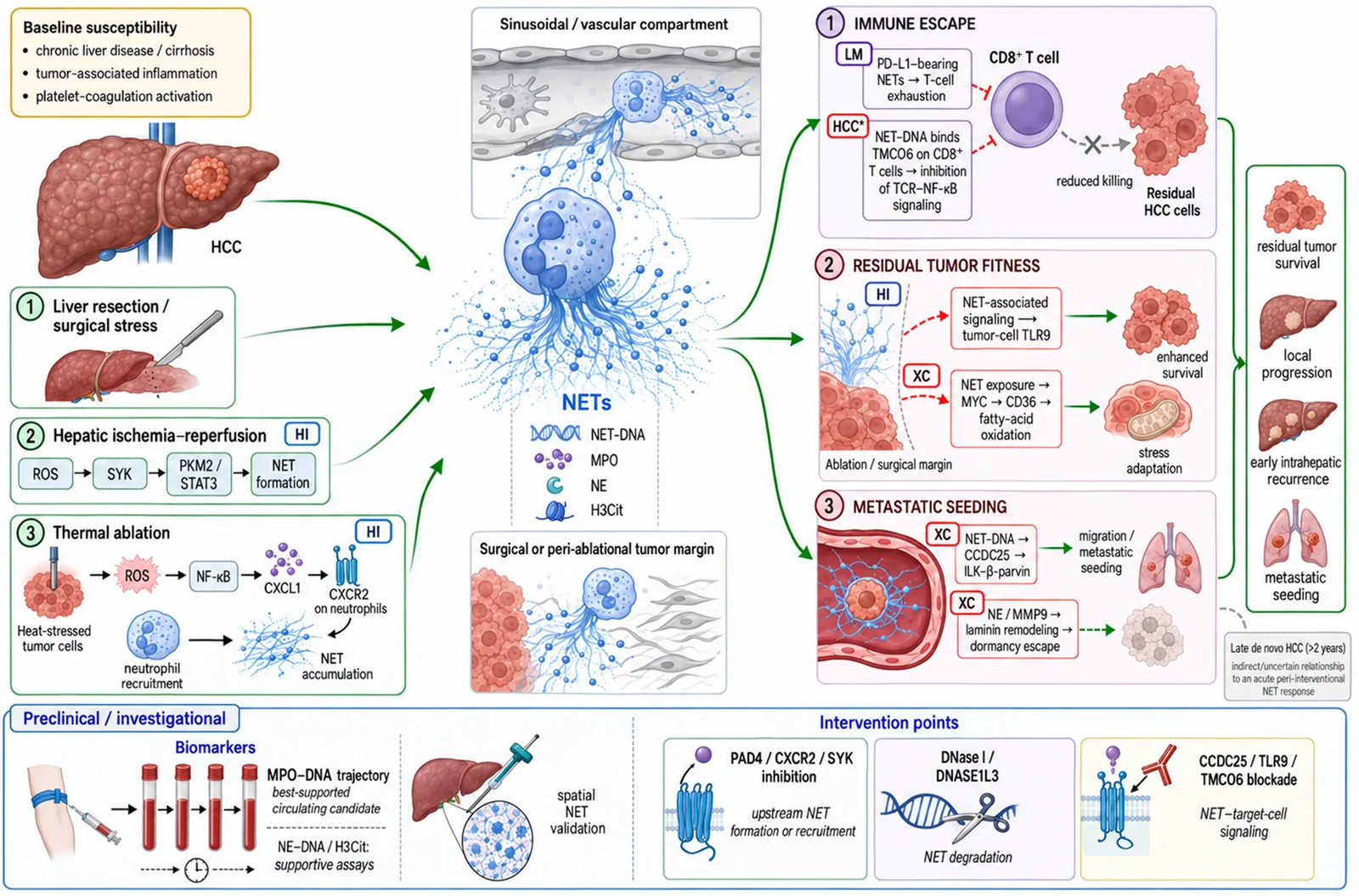
Proposed peri-interventional framework linking liver-directed injury, neutrophil extracellular trap accumulation, immune dysfunction, residual-tumor fitness, and recurrence. Baseline susceptibility may be influenced by chronic liver disease/cirrhosis, tumor-associated inflammation, and platelet–coagulation activation. Resection, hepatic ischemia–reperfusion, and thermal ablation can promote neutrophil recruitment and NET accumulation through partially distinct pathways. Evidence-domain tags placed adjacent to representative pathways denote HCC* (HCC-specific but non-peri-interventional evidence), HI (hepatic-intervention evidence without an HCC-specific oncologic endpoint), LM (liver-metastasis evidence), or XC (cross-cancer mechanistic evidence); these tags indicate source/context rather than formal certainty. Three downstream categories are illustrated: immune escape, residual-tumor fitness, and metastatic seeding. The figure links these mechanisms primarily to residual/local progression, early intrahepatic recurrence, and metastatic dissemination. Late de novo HCC (>2 years) is shown separately because an acute peri-interventional NET response has a weaker and indirect biological relationship to later field carcinogenesis. Representative mechanisms include PD-L1-bearing NETs (LM), NET-DNA–TMCO6 signaling (HCC*), thermal-injury/TLR9 signaling (HI), MYC–CD36–fatty-acid oxidation (XC), NET-DNA–CCDC25 signaling (XC), and NE/MMP9-dependent dormancy escape (XC). The lower panel summarizes investigational biomarkers and intervention points. Serial MPO–DNA is prioritized in the text as the best-supported circulating peri-interventional candidate, with NE–DNA/H3Cit-based measures considered supportive and with spatial NET validation where feasible. All mechanisms and therapeutic targets remain investigational; the figure does not imply that NETs, ablation, or any evidence domain is uniformly tumor promoting.

We conducted a focused narrative search of PubMed/MEDLINE through 2 September 2026; the complete date- and language-bounded query, eligibility criteria, and selection rationale are provided in **Supplementary Methods S1**. Because this Mini Review was not conducted as a prospectively logged systematic review, we do not retrospectively report search-hit or screening-flow counts. For auditability, the final bibliography contains 63 references, including 42 original human or experimental studies and 21 contextual sources (reviews, guidelines/consensus statements, or meta-analyses). **Table 1** presents 10 sentinel original studies selected to represent the principal intervention contexts, evidence domains, recurrence phenotypes, and countervailing findings rather than an exhaustive evidence inventory.

## 2 Resection and thermal ablation remodel the peri-interventional tumor microenvironment

The peri-interventional niche is shaped by more than NETs. Tissue injury simultaneously alters endothelial integrity, coagulation, platelet activation, macrophage signaling, extracellular-matrix structure, antigen release, and lymphocyte access (6, 28). NET accumulation is one component of this treatment-remodeled tumor immune microenvironment, not an isolated linear pathway.

### 2.1 Resection-associated stress and hepatic ischemia-reperfusion

The strongest liver-specific surgical evidence is LM rather than direct peri-interventional HCC evidence. In CRLM resection (LM evidence), higher postoperative MPO–DNA was associated with shorter disease-free survival (14). Liver-intervention and metastasis models linked surgical stress or hepatic ischemia-reperfusion with NET deposition and metastatic growth; DNase I or PAD4-directed perturbation attenuated these effects (14, 29, 30). This evidence does not establish NETs as an independent cause of HCC recurrence: procedural injury and complications can alter NET-associated markers, while recurrence also depends on tumor burden, liver function, and treatment quality (1, 2). Prospective HCC studies need prespecified operative sampling phases and separate analyses of marginal residual disease, *de novo* intrahepatic tumors, and extrahepatic seeding.

Surgical stress may affect processes beyond tumor-cell capture. In XC laparotomy models, NET exposure activated MYC, increased CD36-dependent long-chain fatty-acid uptake and fatty-acid oxidation, and supported anoikis resistance; DNase I, GSK484, PAD4 loss, or metabolic inhibition reduced surgery-enhanced growth (20). In LM evidence from CRLM samples and a hepatic ischemia-reperfusion metastasis model, PD-L1-bearing NETs promoted functional and metabolic T-cell exhaustion, whereas DNase I or perioperative PD-L1 blockade restored T-cell activity experimentally (19). These studies provide mechanistic bridges to immune escape, but neither demonstrates that this pathway drives recurrence after curative-intent HCC resection.

Ischemia-reperfusion amplifies local inflammation during inflow occlusion, partial hepatectomy, and transplantation. Reoxygenation generates reactive oxygen species, activates sinusoidal endothelium, and recruits neutrophils. HI evidence from human transplant samples and experimental transplantation implicates neutrophil CEACAM1-S1PR2/S1PR3 signaling in susceptibility to NET formation and tissue injury (31). In preclinical HI models, tetramethylpyrazine, curcumin, and TLR9-directed hydroxychloroquine reduced NET-associated injury (32–34). These results inform mechanism but do not support perioperative use or recurrence prevention.

Separate HI evidence linked neutrophil SYK to PKM2 nuclear translocation and STAT3 phosphorylation, whereas macrophage SYK amplified NLRP3–IL-1β signaling (15). SYK inhibition reduced NET formation, liver injury, and recurrence in mouse tumor models; neutrophils from patients undergoing portal occlusion supplied a human pathway component but no clinical oncologic endpoint (15). Transcriptomic HI analyses after transplantation add context but cannot confirm extracellular NET structures or NET-mediated recurrence (35). The available data place NETs within a multicellular immune network involving neutrophils, macrophages, sinusoidal endothelium, platelets, and coagulation, rather than a stand-alone pathway (36).

Spatial heterogeneity complicates interpretation. HCC NET biology is itself spatially heterogeneous (12), and hepatic ischemia-reperfusion unfolds within a zonated sinusoidal microenvironment (31, 36). Peripheral assays average these local signals. Total circulating DNA is not NET-specific and may reflect parenchymal injury or altered clearance; liver clearance can materially affect cell-free DNA concentrations during systemic inflammation (37). Mechanistic studies should pair circulating protein–DNA complexes with tissue colocalization and downstream immune or tumor-cell readouts.

### 2.2 Thermal ablation and the peri-ablational niche

Clinical MWA evidence. The strongest patient-linked ablation evidence remains HI rather than HCC-specific oncologic evidence and comes from a retrospective mixed liver-cancer cohort undergoing microwave ablation (MWA) (17). Forty patients (24 primary and 16 secondary liver cancers) were sampled before and 24 h after treatment; MPO–DNA was measured in all paired samples and H3Cit in a smaller subset. MPO–DNA increased after MWA and the fold increase was greater among patients with local tumor progression, but independent prognostic value was not established. Ablation-zone size, treatment completeness, margin adequacy, and tissue-injury burden could influence both NET-associated signals and local progression.

Experimental RFA evidence. The mechanistic arm of the same study used orthotopic radiofrequency ablation (RFA), not MWA, together with patient-derived neutrophils (17). Heat-stressed tumor cells activated ROS–NF-κB–CXCL1/CXCR2 signaling, recruited neutrophils, and generated NET-associated TLR9 signaling with impaired CD8^+^ T-cell responses; NET inhibition or degradation reduced progression experimentally. This modality mismatch limits direct alignment of thermal dose distribution, injury volume, and NET kinetics. A 24-h circulating rise does not establish persistence, so serial sampling is needed.

Insufficient-RFA evidence. The coagulative-necrosis core, sublethal rim, surviving peripheral tumor, and uninvolved liver are biologically distinct compartments; extracellular DNA at the margin should not be labeled as NETs without neutrophil-protein colocalization and exclusion of necrotic debris (38, 39). A separate LM evidence stream concerns insufficient RFA of CRLM: residual tumor cells induced CD177hi/PAD4hi neutrophils and promoted lung metastasis (18). This setting should not be conflated with recurrence after technically complete HCC ablation. Future clinical studies should prespecify ablation modality, treatment completeness, ablation-zone size, delivered injury burden, and minimal ablative margin.

### 2.3 Resection versus thermal ablation: shared and treatment-specific NET contexts

Resection and thermal ablation share DAMP release, oxidative stress, vascular/coagulation activation, and neutrophil recruitment but differ in dominant triggers and spatial organization. Resection combines tissue transection/manipulation with variable ischemia–reperfusion, so HI/LM evidence emphasizes sinusoidal, resection-margin, and early postoperative responses. Thermal ablation creates a coagulative-necrosis core and sublethal rim, where heat stress can activate ROS–NF-κB–CXCL1/CXCR2 signaling; patient-linked data show a 24-h circulating rise but do not define persistence. No head-to-head HCC study has compared NET magnitude, decay, spatial distribution, or immune consequences between procedures.

## 3 Immune and metastatic bridges from net accumulation to residual-disease fitness

As summarized in **Figure 1**, peri-interventional NET accumulation may influence residual-disease survival, early intrahepatic recurrence, and metastatic dissemination through non-mutually exclusive mechanisms. A direct relationship with late *de novo* multicentric HCC is less biologically plausible because a transient treatment-associated inflammatory response is temporally remote from later field-carcinogenesis events.

### 3.1 Vascular retention and extracellular-matrix remodeling

XC evidence indicates that intravascular NETs can retain CTCs under flow and increase hepatic metastatic seeding (7). HCC-specific but non-peri-interventional evidence links NET-producing neutrophils with tumor-cell motility and endothelial disruption (40), while additional XC evidence shows NET-DNA–CCDC25–ILK–β-parvin signaling toward NET-rich organs (41). These mechanisms are most relevant to peri-interventional dissemination and extrahepatic metastasis rather than late *de novo* HCC. Pre-existing CTCs, manipulation-released cells, and locally surviving tumor remain distinct disease substrates.

NET-associated proteases may also remodel tissue niches (42). In XC mouse lung models, NE and matrix metalloproteinase 9 exposed a laminin-derived proliferative signal that awakened dormant disseminated cells (6), providing a candidate mechanism for early micrometastatic reactivation. Whether this occurs in treated liver is unknown and would require spatial demonstration of NET structures, protease-dependent matrix cleavage, and tumor-cell cycle re-entry within the same microregion.

### 3.2 Immune dysfunction and immune escape

HI evidence from thermal-ablation liver models indicates that NET accumulation can impair CD8^+^ T-cell responses (17), whereas LM evidence from CRLM resection and hepatic ischemia-reperfusion links PD-L1-bearing NETs to T-cell exhaustion (19). HCC-specific but non-peri-interventional evidence shows NET-DNA–TMCO6 suppression of CD8^+^ T-cell signaling (22) and cirrhotic-matrix-driven immunosuppressive NET formation with attenuated anti-PD-1 responses (43). XC studies further link NET-rich states to impaired cytotoxicity or T-cell exclusion (44–46), and LM evidence extends this framework to NK-cell surveillance through CCDC25–ILK–STAT3 signaling (21). These pathways are most plausibly relevant to residual disease, early intrahepatic recurrence, and metastatic escape; direct clinical validation after curative-intent HCC treatment is lacking.

Ablation is not uniformly immunosuppressive. In a human HCC cohort, radiofrequency ablation increased tumor-associated-antigen-specific T-cell responses in 62.3% of patients, and post-ablation T-cell responses were associated with recurrence-free outcomes, although the responses were not uniformly durable (23). Tumor-antigen release and priming may coexist with NET-associated immune suppression after ablation (17). The balance is likely to depend on time, compartment, and treatment completeness. NET measurements should be paired with direct assessment of CD8^+^ T-cell localization, activation, clonality, and effector function; NET abundance alone cannot establish immune suppression.

### 3.3 Metabolic adaptation

XC evidence from surgery-induced NET models links MYC–CD36–fatty-acid oxidation to survival under detachment stress (20), and other metastatic models suggest NET-supported mitochondrial homeostasis (47). These pathways may facilitate peri-interventional survival of circulating or residual cells and thus early recurrence, but peri-ablational cells face distinct heat, hypoxia, and nutrient gradients. They remain candidate mechanisms, not validated peri-interventional HCC targets.

### 3.4 Context-dependent and countervailing functions of NETs

NET biology is context dependent rather than uniformly tumor promoting. Its clearest host-protective role is antimicrobial containment; NETs trap bacteria, and PAD4-dependent NET formation contributes to antibacterial defense in selected models (8, 48). NETs also intersect with wound repair (49), but beneficial neutrophil functions should not be equated with beneficial NET effects. CD177^+^ neutrophils promote extracellular-matrix remodeling and HGF-α release during ALPPS-associated liver regeneration (50), but a NET-dependent role is unproven. Conversely, excessive intrahepatic NET accumulation was associated with posthepatectomy liver failure, and DNase I accelerated regeneration in a murine partial-hepatectomy model (16). In LM evidence, NET-associated NE enhanced macrophage phagocytosis and impeded metastatic seeding (51). These findings support context-specific interpretation and caution against indiscriminate peri-interventional NET suppression.

## 4 Net-associated biomarkers: response, prognostic, or predictive?

NET-associated measurements should be defined by intended use. Serial assays after resection or ablation may serve as monitoring biomarkers; pharmacodynamic use requires demonstration of biological response or target engagement during a NET-directed intervention. Prognostic markers must add information beyond liver function, tumor burden, treatment quality, and complications, whereas predictive markers must identify differential treatment benefit. No NET assay is a validated predictive biomarker after curative-intent HCC treatment.

Candidate biomarkers should be ranked by directness of evidence. MPO–DNA currently has the strongest peri-interventional human support, with postoperative associations after CRLM resection and dynamic increases after thermal ablation; separate HCC-specific evidence also links preoperative MPO–DNA to recurrence-related outcomes (14, 17, 52). NE–DNA offers biologically plausible neutrophil attribution but has substantially less direct peri-interventional clinical validation and should be complementary rather than equivalent. H3Cit or H3Cit–DNA provides orthogonal evidence of chromatin citrullination, whereas total cfDNA should not be interpreted as a NET marker in isolation (38, 39, 53).

At minimum, circulating NET-associated activity should include a neutrophil-protein–DNA complex assay, currently preferably MPO–DNA, plus an orthogonal NET/neutrophil readout and standardized pre-analytics. Tissue identification should combine extracellular DNA morphology with MPO or NE colocalization, preferably supported by H3Cit, while excluding necrosis and ablation debris (38, 39). Clinical analyses should prespecify liver function and cirrhosis severity, liver-disease etiology, infection/systemic inflammation, neutrophil count, transfusion and complications, procedural injury, ischemia/resection extent, ablation modality and margin adequacy, and impaired hepatic clearance. H3Cit-DNA is elevated in cirrhosis and HCC and correlates with liver dysfunction without a clear HCC increment over cirrhosis (24).

Compartment is itself a biological variable. Peripheral blood can dilute a concentrated surgical-bed or peri-ablational signal, whereas tissue provides spatial information at few time points. When feasible, blood trajectories should be linked to tumor, adjacent liver, resection-edge, or ablation-margin NET burden and local cytotoxic-lymphocyte phenotypes. Sampling should capture baseline, early post-treatment kinetics, and early follow-up, with invasive portal/hepatic venous sampling or margin biopsy restricted to clinically justified protocols.

## 5 Therapeutic opportunities and safety constraints

Potential interventions act at different levels (54–56). PAD4, CXCR2, or SYK inhibition may reduce NET formation or neutrophil recruitment (14, 15, 17). DNase I has disrupted extracellular DNA scaffolds in perioperative, ablation, dormancy, and metastasis models (6, 14, 17, 19), whereas DNASE1L3-based strategies remain preclinical (44). DNA degradation may leave histones, NE, MPO, and other effectors active; complementary target-cell nodes include CCDC25, TLR9, and TMCO6, with NE/MMP9 and MYC–CD36–fatty-acid oxidation representing downstream pathways (6, 20, 22, 41, 57).

None of these nodes is a clinically validated NET-specific therapy. PAD4, CXCR2, SYK, TLR9, and NF-κB are pleiotropic regulators rather than NET-specific switches (15, 17, 34, 48). Mechanistic development should therefore demonstrate change in a composite NET trajectory or spatial burden, the proposed downstream immune/tumor effect, and preservation of neutrophil number and non-NET functions. This is particularly important for checkpoint combinations, where apparent synergy could reflect broad myeloid suppression rather than NET-specific target engagement (58).

NET–checkpoint combinations remain preclinical hypotheses and must be interpreted against current adjuvant HCC data. The initial RFS signal with atezolizumab–bevacizumab in IMbrave050 was not sustained with longer follow-up, and the updated benefit–risk assessment did not support routine adjuvant use (59). Likewise, phase III KEYNOTE-937 did not demonstrate an RFS benefit with pembrolizumab after curative-intent resection or local ablation (60). Experimental restoration of T-cell function by NET inhibition therefore cannot be equated with postoperative clinical benefit.

Future clinical development should prioritize biomarker-driven early-phase studies. NET-associated measurements are best positioned as exploratory enrichment biomarkers for a high or persistent peri-interventional NET response and as pharmacodynamic biomarkers for target engagement and downstream immune modulation; a predictive designation is not currently supported. Protocols should prespecify recurrence phenotype, treatment completeness, ischemia time or ablation margin, liver-disease etiology, infection, transfusion, concomitant immunotherapy, cytotoxic-lymphocyte readouts, and safety outcomes including infection, liver regeneration, wound healing, bleeding, and thrombosis.

## 6 Discussion and translational priorities

Evidence for the peri-interventional framework remains uneven: resection data are dominated by CRLM, patient-linked ablation data derive from a mixed liver-malignancy cohort, and human ischemia-reperfusion studies generally lack oncologic endpoints (14, 15, 17, 19, 31, 35). Adequate versus insufficient ablation and local residual tumor versus occult micrometastases, dormant cells, and CTCs must remain separate analytical settings. The framework therefore rests on mechanistic coherence rather than clinical validation.

Whether NETs mediate recurrence-relevant biology or simply mark treatment injury remains unresolved. Causal interpretation requires temporal precedence, spatial correspondence, a composite NET definition, and perturbation of the proposed downstream mechanism. For cancer-immunity questions, direct CD8^+^/NK-cell localization, activation, exhaustion, and cytotoxic function are essential. Critically, a transient peri-interventional NET peak is biologically better aligned with residual disease, early intrahepatic recurrence, or metastatic dissemination than with late *de novo* HCC.

Peripheral biomarkers cannot substitute for spatial assessment of the local tumor or treatment-bed immune microenvironment. In resected early-stage HCC, high capsular lymphatic vessel density was associated with worse postoperative outcomes and adverse pathological features (61), consistent with meta-analytic hepatobiliary evidence (62). Conversely, host immune state also extends beyond local tissue: HCC in compensated viral cirrhosis was associated with reduced CT-derived thymic dimensions after propensity-score matching (63). These complementary data support a multi-compartment approach integrating serial blood measurements, spatial tissue evidence, and broader host immune phenotyping.

Treatment-associated NET accumulation is a testable component of the peri-interventional tumor immune microenvironment, not an established cause of HCC recurrence. The immediate priority is an intervention- and recurrence-phenotype-stratified prospective study that defines NET kinetics, validates circulating measures against spatial evidence, and links NET dynamics to cytotoxic lymphocyte function. NET-targeting trials, alone or with checkpoint blockade, should proceed only after prognostic value, target engagement, and safety are established.
