# Supplementary material for: Peri-interventional neutrophil extracellular traps and immune surveillance in hepatocellular carcinoma: mechanisms, biomarkers, and recurrence after liver resection or thermal ablation
Source: Front Oncol. 2026 Sep 28;16:1988923. doi: 10.3389/fonc.2026.1988923 (PMC13655784; doi:10.3389/fonc.2026.1988923)
Supplement: Supplementary file 1 [file Table1.docx]

**Supplementary Methods S1. Detailed literature-search strategy**

**Database, search date, and scope.** PubMed/MEDLINE was searched for literature within the scope of this focused narrative Mini Review, with the search specified through 2 September 2026. The documented query below includes an explicit publication-date cutoff and English-language field restriction. Reference lists of relevant original studies and reviews were also screened for additional directly relevant reports.

**Exact PubMed/MEDLINE query.** ("Neutrophil Extracellular Traps"[Mesh] OR "neutrophil extracellular trap"[Title/Abstract] OR "neutrophil extracellular traps"[Title/Abstract] OR NETosis[Title/Abstract]) AND ("Carcinoma, Hepatocellular"[Mesh] OR "hepatocellular carcinoma"[Title/Abstract] OR HCC[Title/Abstract] OR liver[Title/Abstract] OR hepatic[Title/Abstract]) AND (resection[Title/Abstract] OR hepatectomy[Title/Abstract] OR surgery[Title/Abstract] OR "ischemia-reperfusion"[Title/Abstract] OR ablation[Title/Abstract] OR radiofrequency[Title/Abstract] OR microwave[Title/Abstract] OR recurrence[Title/Abstract] OR metastas*[Title/Abstract] OR biomarker*[Title/Abstract] OR "T cell"[Title/Abstract] OR "NK cell"[Title/Abstract] OR immunotherap*[Title/Abstract]) AND English[la] AND 1900/01/01:2026/09/02[dp]

**Eligibility and use of evidence.** We considered peer-reviewed original human or experimental studies that (i) directly evaluated intervention-associated NET formation, release, accumulation, or NET-associated biomarkers in HCC or a hepatic intervention; (ii) provided a mechanistically necessary liver-metastasis or cross-cancer bridge for pathways discussed in the review; or (iii) provided clinically relevant HCC context required to interpret recurrence phenotypes or the contemporary adjuvant-immunotherapy setting. Reviews, guidelines, consensus statements, and meta-analyses were used for background, assay interpretation, or clinical context rather than as primary mechanistic evidence. Studies based only on nonspecific inflammation or total cfDNA without NET-directed evidence were not treated as direct NET evidence. Cross-cancer studies were used only when an otherwise unavailable mechanism was directly relevant to peri-interventional immune surveillance, residual-disease fitness, or metastatic seeding.

**Bibliography audit and Table 1 selection.** Because this work is a focused narrative Mini Review rather than a prospectively logged systematic review, we do not retrospectively report search-hit, screening, or PRISMA-style inclusion counts. For an auditable account of the evidence actually used, the final manuscript bibliography contains 63 references: 42 original human or experimental studies and 21 contextual sources comprising reviews, guidelines/consensus statements, or meta-analyses. These are bibliography counts and should not be interpreted as search-derived screening counts. Table 1 summarizes 10 sentinel original studies selected to span resection/surgical stress, hepatic ischemia-reperfusion, posthepatectomy regeneration, thermal ablation, insufficient ablation, T-cell and NK-cell surveillance, metabolic adaptation, HCC-specific NET biology, and a countervailing post-RFA immune response. Selection favored studies with a human component, direct perturbation of NET biology, or a uniquely informative mechanistic bridge; redundant studies addressing the same mechanism were not all tabulated.

**Reproducibility note.** The query is reported verbatim with explicit language and publication-date limits so that its intended scope can be rerun. PubMed records can be added or updated retrospectively; therefore, a later rerun may not reproduce the historical result count exactly. For this reason, no retrospective historical search-result count is asserted.
